# Supplementary material for: Evolutionary analysis of endogenous intronic retroviruses in primates reveals an enrichment in transcription binding sites associated with key regulatory processes
Source: PeerJ. 2022 Dec 22;10:e14431. doi: 10.7717/peerj.14431 (PMC9790151; doi:10.7717/peerj.14431)
Supplement: File S1 [file peerj-10-14431-s001.pdf]

Table S1. Genomic locations of *de novo* predicted ERVs in humans.

	Intragenic ERVs	Non-intragenic ERVs	
**ERV location**	**Gene name**	**Adjacent genes**	
Chr19:44862867-44869439	NECTIN-2		
Chr20:37149869-37161833	MROH8		
Chr9:128894025-128897010	LRRC8A		
Chr1:211835983-211845425	LPGAT1-AS1		
Chr2:186073944-186078649	LINC01473		
Chr5:61205536-61207819	SMIM15-AS1		
Chr1:216032484-216038211	USH2A		
Chr2:46903003-46905220	MCFD2		
Chr3:18172037-18174273	TBC1D5		
Chr3:6661070-6663698	GRM7-AS3		
Chr4:165282824-165285097	KLHL2		
Chr7:116004827-116007089	TFEC		
Chr1:37907815-37914173	INPP5B		
Chr6:94553915-94559610	ENSG00000289178		
Chr11:14524261-14529062	PSMA1		
Chr1:206052519-206058912	RHEX		
Chr6:74468144-74474614	ENSG00000223786		
Chr20:15979869-15986246	MACROD2		
Chr20:15147683-15153522	MACROD2		
Chr4:88538134-88546230	DET1		
Chr8:105619254-105621894	ZFPM2		
Chr3.12:125890459-125899596	FAM86JP		
Chr10:6824176-6833641	LINC00707		
Chr14:67706311-67714076	GPHN		
Chr9:74515385-74517144	RORB		
Chr16:70338524-70347966	DDX19A		
Chr14:77667816-77669413	COX6CP11		
NT_187586.1:200769-203163	DEAF1		
Chr3:45076355-45078647		CLEC3B	CDCP1
Chr4:98703629-98705957		TSPAN5	EIF4E
Chr10:6379439-6381695		DPPA5P3	PRKCQ
Chr13:103267226-103269529		SLC10A2	ATP6V1G1P7
ChrX:123757939-123760219		THOC2	RNU7-69P
ChrX:46170451-46172772		PCNAP3	ACTBP1
ChrX:57639022-57641324		ZXDB	NLRP2B
Chr1:65643203-65648034		LEPR	PDE4B
Chr3:27549571-27553803		SLC4A7	LINC01980
Chr3:67037862-67044184		KBTBD8	SUCLG2
Chr8:115212253-115217982		TRPS1	CARSP2
ChrX:111543805-111549673		SERTM2	EIF4BP7
NT_187675.1:264534-270837		FCAR	NCR1
NT_187676.1:170245-176548		FCAR	NCR1
NT_187683.1:168513-174816		FCAR	NCR1
Chr9:86360724-86367962		ZCCHC6	

Chr6:28766179-28776541		TRA-TGC6-1	
NT_113891.3:256873-267245		TRIM27	
NW_003871091.1:114309-128756		KRT40	KRT39
Chr11:118721015-118730174		TREH	DDX6
Chr1:213025480-213032442		RPS6KC1	ANGEL2
Chr2:152273628-152279762		LOC105373689	FMNL2
Chr8:52979990-52984068		NPBWR1	OPRK1
Chr19:9711509-9717966		ZNF562	ZNF846
Chr2:40671993-40678381		SLC8A1	
Chr3:144903447-144910073		GM2AP1	
Chr10:37275132-37284840		ANKRD30A	TMEM161BP1
Chr10:85184306-85190827		CCSER2	GRID1
Chr11:35047926-35050201		PDHX	CD44
Chr1:38425114-38430783		POU3F1	RRAGC
Chr16:35267618-35277076		ENSG00000260958	
Chr3:91379068-91392888		ENSG00000283367	

***Table S2.* Summary of RetroTector analysis and alignments between INPP5B orthologous vs ERV region in the human gene**. The species found in the table correspond to primates; the species highlighted in purple are those that were detected in RetroTector and BLASTN.

**INPP5B**					
**N°**	**Species**	**RetroTector**	**BLASTN**		
			**Query coverage**	**e-value**	**% identity**
1	*Carlito syrichta*	+	4%	3.00E-20	82.41%
2	*Cebus capucinus imitator*	-	4%	1.00E-72	83.17%
3	*Aotus nancymaae*	-	4%	5.00E-53	79.48%
4	*Saimiri boliviensis*	-	7%	9.00E-80	86.11%
5	*Nomascus leucogenys*	+	97%	0	95.40%
6	*Pongo abelii*	-	84%	0	88.90%
7	*Gorilla gorilla*	+	97%	0	97.43%
8	*Pan troglodytes*	+	97%	0	98.98%
9	*Pan paniscus*	+	97%	0	98.85%
10	*Rhinopithecus roxellana*	+	97%	0	91.84%
11	*Rhinopithecus bieti*	-	83%	0	91.63%
12	*Colobus angolensis palliatus*	+	89%	0	91.40%

13	*Piliocolobus tephrosceles*	-	96%	0	91.51%
14	*Chlorocebus sabaeus*	+	97%	0	89.69%
15	*Cercocebus atys*	+	97%	0	90.40%
16	*Mandrillus leucophaeus*	+	93%	0	90.47%
17	*Papio anubis*	-	4%	2.00E-83	84.47%
18	*Theropithecus gelada*	-	97%	0	90.34%
19	*Macaca fascicularis*	-	100%	0	90.93%
20	*Macaca nemestrina*	+	93%	0	90.62%
21	*Macaca mulatta*	-	25%	0	89.91%
22	*Otolemur garnetti*	-	4%	6.00E-20	76.72%
23	*Propithecus coquereli*	-	-		
24	*Microcebus murinus*	-	6%	5.00E-43	77.46%

***Table S3.* Summary of RetroTector analysis and alignments between DET1 orthologous vs ERV region in the human gene.** The species found in the table correspond to primates; the species highlighted in purple are those that were detected in RetroTector and BLASTN.

**DET1**					
**N°**	**Species**	**RetroTector**	**BLASTN**		
			**Query coverage**	**e-value**	**% identity**
1	*Carlito syrichta*	-	-		
2	*Cebus capucinus imitator*	-	-		
3	*Aotus nancymaae*	-	-		
4	*Saimiri boliviensis*	-	-		
5	*Nomascus leucogenys*	+	88%	0	93.92%
6	*Pongo abelii*	+	88%	0	93.66%
7	*Gorilla gorilla*	+	92%	0	97.34%
8	*Pan troglodytes*	+	92%	0	98.51%
9	*Pan paniscus*	+	92%	0	98.55%
10	*Rhinopithecus roxellana*	-	23%	0.00	82.69%

11	*Rhinopithecus bieti*	-	-		
12	*Colobus angolensis palliatus*	-	6%	4.00E-101	79.89%
13	*Piliocolobus tephrosceles*	-	-		
14	*Chlorocebus sabaeus*	-	12%	7.00E-175	78.97%
15	*Cercocebus atys*	-	8%	3.00E-118	77.98%
16	*Mandrillus leucophaeus*	-	11%	2.00E-136	76.68%
17	*Papio anubis*	-	23%	0	82.84%
18	*Theropithecus gelada*	-	23%	0	83.82%
19	*Macaca fascicularis*	-	23%	0	82.56%
20	*Macaca nemestrina*	-	11%	1.00E-97	76.04%
21	*Macaca mulatta*	-	23%	0	82.76%
22	*Otolemur garnetti*	-	-		
23	*Propithecus coquereli*	-	-		
24	*Microcebus murinus*	-	-		

***Table S4.* Summary of RetroTector analysis and alignments between PSMA1 orthologous vs ERV region in the human gene.** The species found in the table correspond to primates; the species highlighted in purple are those that were detected in RetroTector and BLASTN.

PSMA1					
**N°**	**Species**	**RetroTector**	**BLASTN**		
			**Query coverage**	**e-value**	**% identity**
1	*Carlito syrichta*	-	-		
2	*Cebus capucinus imitator*	-	-		
3	*Aotus nancymaae*	-	-		
4	*Saimiri boliviensis*	-	-		
5	*Nomascus leucogenys*	-	52%	0	95.84%
6	*Pongo abelii*	-	-		
7	*Gorilla gorilla*	+	100%	0	97.88%
8	*Pan troglodytes*	+	100%	0	98.38%

9	*Pan paniscus*	+	100%	0	98.40%
10	*Rhinopithecus roxellana*	-	-		
11	*Rhinopithecus bieti*	-	-		
12	*Colobus angolensis palliatus*	+	-		
13	*Piliocolobus tephrosceles*	-	-		
14	*Chlorocebus sabaeus*	-	-		
15	*Cercocebus atys*	-	-		
16	*Mandrillus leucophaeus*	-	-		
17	*Papio anubis*	-	-		
18	*Theropithecus gelada*	-	-		
19	*Macaca fascicularis*	+	-		
20	*Macaca nemestrina*	-	-		
21	*Macaca mulatta*	-	-		
22	*Otolemur garnetti*	-	-		
23	*Propithecus coquereli*	-	-		
24	*Microcebus murinus*	-	-		

***Table S5*. Summary of RetroTector analysis and alignments between USH2A orthologous vs ERV region in the human gene.** The species found in the table correspond to primates; the species highlighted in purple are those that were detected in RetroTector and BLASTN

**USH2A**					
**N°**	**Species**	**RetroTector**	**BLASTN**		
			**Query coverage**	**e-value**	**% identity**
1	*Carlito syrichta*	-	-		
2	*Cebus capucinus imitator*	-	45%	0	88.43%
3	*Aotus nancymaae*	-	47%	0	88.42%
4	*Saimiri boliviensis*	-	39%	0	87.11%
5	*Nomascus leucogenys*	+	100%	0	95.91%

6	*Pongo abelii*	+	52%	0	95.78%
7	*Gorilla gorilla*	+	100%	0	98.15%
8	*Pan troglodytes*	+	100%	0	98.66%
9	*Pan paniscus*	+	100%	0	98.52%
10	*Rhinopithecus roxellana*	-	98%	0	91.19%
11	*Rhinopithecus bieti*	-	98%	0	91.10%
12	*Colobus angolensis palliatus*	-	98%	0	91.88%
13	*Piliocolobus tephrosceles*	+	98%	0	91.39%
14	*Chlorocebus sabaeus*	-	98%	0	91.59%
15	*Cercocebus atys*	-	98%	0	91.53%
16	*Mandrillus leucophaeus*	-	98%	0	91.20%
17	*Papio anubis*	-	98%	0	91.51%
18	*Theropithecus gelada*	-	98%	0	91.29%
19	*Macaca fascicularis*	-	98%	0	91.28%
20	*Macaca nemestrina*	-	98%	0	91.29%
21	*Macaca mulatta*	-	98%	0	91.23%
22	*Otolemur garnetti*	+	11%	2.00E-40	72.99%
23	*Propithecus coquereli*	+	-		
24	*Microcebus murinus*	+	6%	3.00E-49	77.44%

***Table S6.* Summary of RetroTector analysis and alignments between MACROD2 orthologous vs ERV1 region in the human gene.** The species found in the table correspond to primates; the species highlighted in purple are those that were detected in RetroTector and BLASTN

MACROD2-ERV1					
**N°**	**Species**	**RetroTector**	**BLASTN**		
			**Query coverage**	**e-value**	**% identity**
1	*Carlito syrichta*	-	-		
2	*Cebus capucinus imitator*	+	-		
3	*Aotus nancymaae*	+	-		

4	*Saimiri boliviensis*	-	-		
5	*Nomascus leucogenys*	+	100%	0	94.95%
6	*Pongo abelii*	+	15%	0	95.42%
7	*Gorilla gorilla*	+	100%	0	97.60%
8	*Pan troglodytes*	+	100%	0	98.39%
9	*Pan paniscus*	+	100%	0	97.96%
10	*Rhinopithecus roxellana*	+	100%	0	80.71%
11	*Rhinopithecus bieti*	+	55%	0	81.07%
12	*Colobus angolensis palliatus*	+	85%	0	84.49%
13	*Piliocolobus tephrosceles*	+	100%	0	80.11%
14	*Chlorocebus sabaeus*	+	-		
15	*Cercocebus atys*	-	95%	0	81.99%
16	*Mandrillus leucophaeus*	+	-		
17	*Papio anubis*	+	100%	0	81.78%
18	*Theropithecus gelada*	+	100%	0	82.04%
19	*Macaca fascicularis*	-	100%	0	81.51%
20	*Macaca nemestrina*	+	26%	4.00E-154	81.47%
21	*Macaca mulatta*	+	100%	0	81.51%
22	*Otolemur garnetti*	-	-		
23	*Propithecus coquereli*	-	-		
24	*Microcebus murinus*	-	-		

Table S7. **Summary of RetroTector analysis and alignments between MACROD2 orthologous vs ERV2 region in the human gene.** The species found in the table correspond to primates; the species highlighted in purple are those that were detected in RetroTector and BLASTN

**MACROD2-ERV2**					
**N°**	**Species**	**RetroTector**	**BLASTN**		
			**Query coverage**	**e-value**	**% identity**
1	*Carlito syrichta*	-	-		

2	*Cebus capucinus imitator*	+	-		
3	*Aotus nancymaae*	+	-		
4	*Saimiri boliviensis*	-	-		
5	*Nomascus leucogenys*	+	-		
6	*Pongo abelii*	+	100%	0	94%
7	*Gorilla gorilla*	+	-		
8	*Pan troglodytes*	+	100%	0	97.91%
9	*Pan paniscus*	+	100%	0	97.91%
10	*Rhinopithecus roxellana*	+	12%	5.00E-91	88.24%
11	*Rhinopithecus bieti*	-	-		
12	*Colobus angolensis palliatus*	+	9%	4.00E-87	87.50%
13	*Piliocolobus tephrosceles*	+	9%	1.00E-87	87.50%
14	*Chlorocebus sabaeus*	+	9%	1.00E-87	87.50%
15	*Cercocebus atys*	-	-		
16	*Mandrillus leucophaeus*	+	9%	1.00E-91	88.28%
17	*Papio anubis*	+	9%	8.00E-74	84.00%
18	*Theropithecus gelada*	+	11%	5.00E-91	88.24%
19	*Macaca fascicularis*	-	9%	1.00E-87	87.54%
20	*Macaca nemestrina*	+	9%	1.00E-91	88.28%
21	*Macaca mulatta*	+	9%	3.00E-88	87.59%
22	*Otolemur garnetti*	-	-		
23	*Propithecus coquereli*	+	-		
24	*Microcebus murinus*	-	-		

***Table S8.* Summary of RetroTector analysis and alignments between INPP5B paralogous vs ERV region in the human gene.** Only one positive result in RetroTector was found in SYNJ1; however, according to the alignment result, it is not the same retroviral sequence. Therefore, it is a different ERV to the one found in the INPP5B gene.

**Paralogues**	**RetroTector**	**BLASTN**

OCRL	-	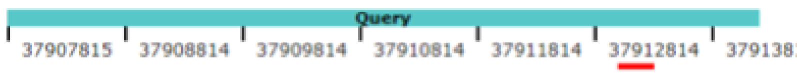
SYNJ1	+	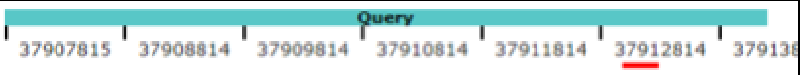
SYNJ2	-	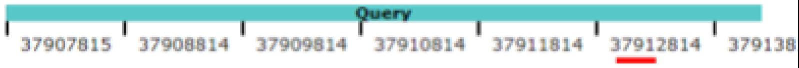
INPP5J	-	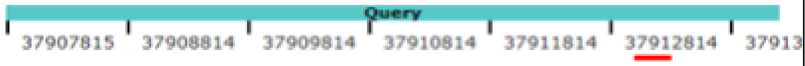
INPP5D	-	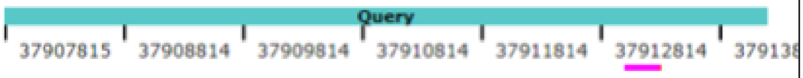
INPP5F	-	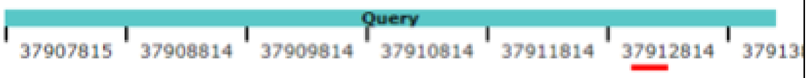
INPP5K	-	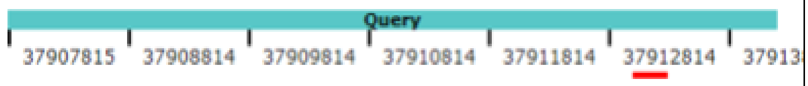
FIG4	-	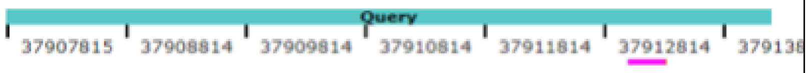
SACM1L	-	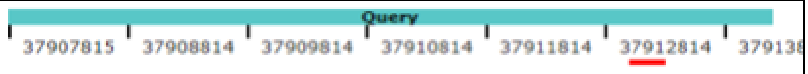
SH2D1A	-	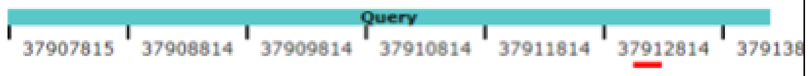
SH2D1B	-	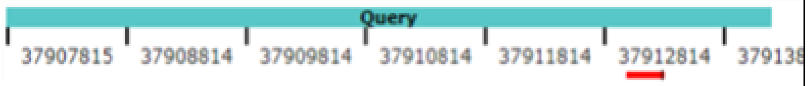

Table S9. **Summary of RetroTector analysis and alignments between USH2A paralogous vs ERV region in the human gene.** Only two positive results in RetroTector were found in LAMA2 and NTN4; however, according to the alignment result, it is not the same retroviral sequence. Therefore, it is a different ERV to the one found in the USH2A gene.

Paralogues	RetroTector	BLASTN
LAMA1	-	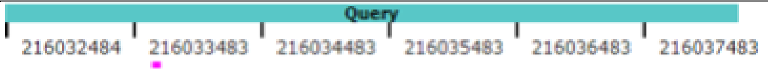
NTNG2	-	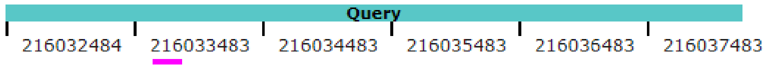
HSPG2	-	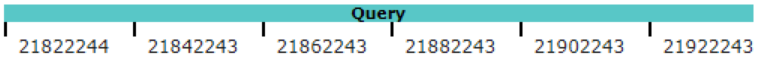
ATRN	-	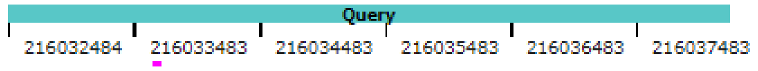
EGFLAM	-	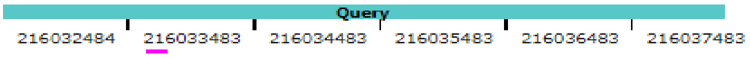
MSANTD3-TMEFF1	-	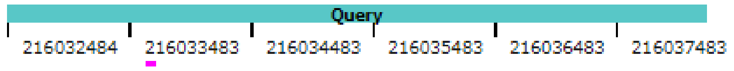
TMEFF1	-	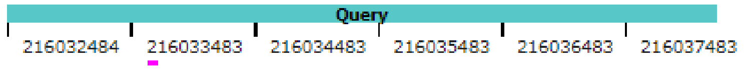
LAMA4	-	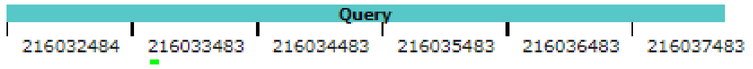

LAMA2	+	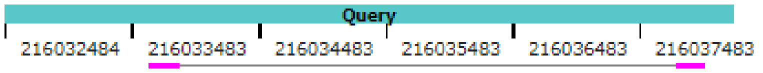
NTN4	+	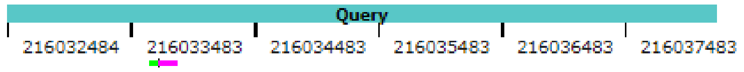
MEGF9	-	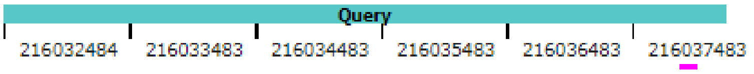
LAMA3	-	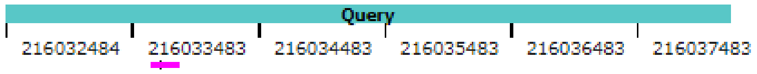
NTN1	-	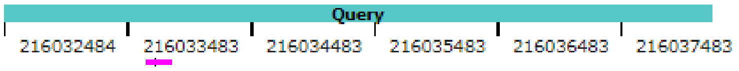

Table S10. Somatic variants associated with tumors along the sections annotated as ERVs.

**Cosmic variant ID***	**Phenotype**
**INPP5B**	
COSV65959311	Esophageal tumor
COSV65959639	Breast tumor
COSV65959760	Breast tumor
COSV65960118	Pancreatic tumor
COSV65960733	Liver tumor
COSV65960967	Hematopoietic and lymphoid tissue tumor
COSV65961219	Liver tumor
COSV65961269	Hematopoietic and lymphoid tissue tumor
COSV65961279	Ovarian tumor
COSV65961492	Ovarian tumor
COSV65961588	Breast tumor
COSV65961603	Hematopoietic and lymphoid tissue tumor
COSV65961705	Liver tumor
COSV65961718	Liver tumor
COSV65961823	Ovarian tumor
COSV65962214	Liver tumor
COSV65962405	Hematopoietic and lymphoid tissue tumor
COSV65962417	Prostate tumor
COSV65962483	Liver tumor
COSV65962514	Liver tumor
COSV65962520	Ovarian tumor
COSV65962976	Ovarian tumor
COSV65963068	Ovarian tumor
COSV65963313	Prostate tumor
COSV65963339	Kidney tumor
**DET1**	
COSV51550681	Hematopoietic and lymphoid tissue tumor

COSV51550823	Upper aerodigestive tract tumor
COSV51550973	Esophageal tumor
COSV51551595	Prostate tumor
COSV51551696	Breast tumor
COSV51551865	Liver tumor
COSV51551976	Kidney tumor
COSV51551985	Kidney tumor
COSV51552255	Breast tumor
COSV51553355	Hematopoietic and lymphoid tissue tumor
COSV51553727	Central nervous system tumor
**PSMA1**	
COSV67166382	Breast tumor
COSV67166400	Stomach tumor
COSV67166463	Breast tumor
COSV67166513	Prostate tumor
COSV67166536	Liver tumor
COSV67166564	Prostate tumor
COSV69859392	Breast tumor
COSV69860027	Breast tumor
COSV69860372	Hematopoietic and lymphoid tissue tumor
COSV69860374	Hematopoietic and lymphoid tissue tumor
COSV69860495	Large intestine tumor
COSV69860859	Hematopoietic and lymphoid tissue tumor
COSV69860862	Hematopoietic and lymphoid tissue tumor
COSV69860943	Hematopoietic and lymphoid tissue tumor
COSV69861018	Hematopoietic and lymphoid tissue tumor
COSV69861108	Esophageal tumor
COSV69861163	Breast tumor
**MACROD2-ERV1**	
COSV54063250	Kidney tumor

*COSMIC database (https://cancer.sanger.ac.uk/cosmic)

Table S11. Transcription factors (TF) associated exclusively with ERVs, excluding all TFs shared by orthologs and paralogs annotated in equivalent structural sections.

INPP5B	***DET1***	***PSMA1***	***USH2A***	***ERV1 MACROD2***	***ERV2 MACROD2***
A4	AFF4	AMH	AR	AGO1	AR
AR	AGO2	ASCL1	BRD4	AHR	BHLHE40
ASCL1	AHR	BMI1	CEBPA	AR	BRD2
BMI1	AR	CBFA2T2	CEBPB	ARID3A	CEBPB
BRD4	ARID1A	CBFA2T3	CTCF	ARNT	CREB1
BRD9	ARNT	CEBPA	DUX4	BATF	CTCF

CASZ1	ASCL1	CEBPB	ELK3	BATF3	CUL4A
CBX3	ATF3	CTCF	ESR1	BCL11A	DTL
CBX5	BMI1	CUL4A	HNF4A	BCL6	DUX4
CEBPA	BRD2	DTL	KLF11	BMI1	EP300
CLOCK	BRD3	DUX4	KLF4	BRD2	ESR1
CTCF	BRD4	ERCC2	KMT2A	BRD4	FOXA1
CUL4A	CAVIN1	ESR1	MYC	CDK9	FOXA2
DCP1A	CBFA2T3	FOSB	NFKB1	CDX2	GATA1
DUX4	CDK8	FOXA2	NUP98	CEBPA	GATA2
EGR2	CDK9	GABPA	PADI2	CEBPB	GATA3
EGR3	CEBPA	GATA6	PPARG	CEBPG	GATA4
ELK3	CEBPB	GTF3C2	RAD21	CENPA	JUND
EP300	CHD1	HDAC2	RELA	CTCF	KLF4
ESR1	CHD4	HIF1A	RING1	DUX4	MAFK
ETS1	CHD7	IKZF1	PHOX2B	E2F1	MBD4
EZH2	CREB1	INO80	RUNX1	E2F8	MCM7
FANCD2	CREB3	IRF4	STAT1	EBF1	MYC
FOXA1	CTCF	JUN	SUZ12	EED	MYOG
FOXA2	DDX20	LDB1	TAL1	ELF1	NEUROD1
FOXO1	DPF2	LMNA	TP63	ELF3	NF2L2
GATA2	E2F6	MAX	TRIM28	EP300	NFATC3
HDAC1	EED	MAZ	UPF1	EPAS1	NR3C1
HDAC2	ELF1	MBD4	YAP1	ERG	OTX2
HIF1A	EOMES	MCM7	ZNF585B	ESR1	PGR
HNF4A	EP300	MITF	ZNF586	ETS1	PPARG
IRF4	ERCC2	MYOD1		FOXA1	RAD21
JUN	ERCC3	MYOG		FOXA2	SMAD2
KLF4	ERCC6	NCOR1		GATA2	SMAD4
KMT2A	ERG	NFE2		GTF3C2	SMARCA4
MBD4	ESR1	NUP98		HDAC1	SMARCC1
MBL2	ETS1	PEX2		HIF1A	SNAI2
MCM7	ETS2	RAD21		HMGA1	SPI1
MECP2	ETV2	SMARCA4		HNF4A	TAL1
MSC	EZH2	SOX17		HOXB13	TLE3
MTOR	FEZF1	SPI1		HOXC9	TRIM28
MYC	FLI1	SSRP1		HSF1	WT1
MYCN	FOS	TAL1		IKZF1	ZBTB7A
MYOD1	FOSB	TBP		IRF4	
MYOG	FOSL1	TBX21		KDM6A	
NCOR2	FOSL2	TRIM28		KLF4	
NFKB1	FOXA1	UPF1		KMT2A	
NFKBIA	FOXA2	WT1		LMNA	
NR3C1	FOXK2	ZNF316		LMNB1	
PADI2	FOXO1	ZNF600		MBD4	
PALB2	GATA1			MBL2	
PARP1	GATA2			MECP2	

PCBP2	GATA3			MEF2B	
POU5F1	GATA4			MLLT1	
PPARG	GATA6			MXD3	
RAD21	GATAD2A			MYC	
RBBP4	GATAD2B			MYCN	
RELA	GLIS1			MYOD1	
RING1	GTF2A2			MYOG	
RNF2	GTF3C2			NFATC3	
RUNX1	HDAC1			NFKB1	
SETDB1	HDAC2			NFKB2	
SIRT1	HDGFL2			NIPBL	
SOX17	HEY1			NOTCH1	
SP5	HINFP			PAX5	
SPI1	HMBOX1			PBX2	
STAT1	HOXB13			PBX3	
SUMO2	HSF1			PGR	
SUPT5H	ID3			PKNOX1	
SUZ12	IKZF1			PPARG	
TCF7L2	IKZF3			PTBP1	
TEAD4	ILK			RAD21	
TFAP2C	INTS11			RBBP5	
TP63	IRF1			RELA	
TRIM24	IRF9			RELB	
TRIM28	JUN			REST	
TRPS1	JUNB			RING1	
WT1	JUND			RUNX3	
ZBTB17	KDM1A			SAP130	
ZNF263	KDM5B			SATB1	
ZNF280A	KLF4			SIN3A	
ZNF467	KMT2A			SKIL	
ZNF600	KMT2B			SMC1A	
ZNF83	KMT2C			SOX8	
ZSCAN29	LMNA			SPI1	
	MAX			SRF	
	MAZ			SSRP1	
	MBD4			STAG1	
	MECOM			STAT3	
	MED12			SUMO2	
	MEF2A			SUZ12	
	MEF2B			TAL1	
	MEF2D			TP53BP1	
	MEIS2			TP63	
	MITF			TRIM22	
	MNT			TRIM28	
	MYC			TRPS1	
	MYCN			UPF1	

	MYNN			WT1	
	MYOD1			XBP1	
	MYOG			YY1	
	NANOG			ZBTB17	
	NCOR1			ZBTB7A	
	NEUROD1			ZFX	
	NFE2			ZSCAN5A	
	NFRKB				
	NKX2-1				
	NR2F1				
	NR2F2				
	NR3C1				
	NR4A1				
	NRC1				
	NSD2				
	NUP98				
	ORC2				
	OSR2				
	OTX2				
	PADI2				
	PDX1				
	PGR				
	PHF8				
	PML				
	POU5F1				
	PPARG				
	PRDM9				
	PRDMI4				
	PTTG1				
	PYGO2				
	RAD21				
	RBBP5				
	RBM22				
	RBM39				
	RCOR1				
	RELA				
	RFX1				
	RING1				
	RMF2				
	RNF2				
	RUNX1				
	RXRA				
	SALL3				
	SCRT1				
	SFPQ				
	SIN3A				

	SMAD1				
	SMAD2				
	SMARCA4				
	SMARCB1				
	SNAI2				
	SOX17				
	SOX2				
	SOX8				
	SP1				
	SPF2				
	SPI1				
	SPIB				
	SRF				
	SRSF3				
	SS18				
	SSRP1				
	STAT5A				
	STAT5B				
	SUMO2				
	SUPT5H				
	SUPT6H				
	TAF1				
	TAL1				
	TBL1XR1				
	TBP				
	TCF12				
	TCF4				
	TEAD4				
	TET2				
	TFAP2C				
	TP53				
	TP63				
	TRIM28				
	TRPS1				
	VEZF1				
	WT1				
	XRCC5				
	YAP1				
	YY1				
	ZBTB40				
	ZBTB48				
	ZBTB6				
	ZBTB7A				
	ZFP91				
	ZFX				
	ZNF574				

	ZNF143				
	ZNF316				
	ZNF329				
	ZNF384				
	ZNF395				
	ZNF639				
	ZNF652				
	ZNF687				
	ZNF740				
	ZNF766				
	ZNF816				
	ZSCAN5A				
	ZSCAN16				

Table S12. Transcription factor enrichment analysis in structural equivalent sections to paralog genes to ERV candidates.

**Gene name**	**N° of unique transcription factors in the equivalent area of the ERV**	**Pathways**	**Transcription factors involved in the Pathway**	**Benjamini***
**INPP5B paralogs**				
SH2D1A	9	Th1 and Th2 cell differentiation	2	1.00E+00
		Progesterone-mediated oocyte maturation	2	1.00E+00
		Oocyte meiosis	2	1.00E+00
		Epstein-Barr virus infection	2	1.00E+00
INPP5D	133	Transcriptional misregulation in cancer	17	1.20E-09
		Chronic myeloid leukemia	8	2.30E-04
		Th17 cell differentiation	9	2.30E-04
		Viral carcinogenesis	11	4.40E-04
		Cell cycle	9	4.40E-04
		Human T-cell leukemia virus 1 infection	11	6.10E-04
		Pathways in cancer	16	1.20E-03
		Hepatitis B	9	1.70E-03

		Th1 and Th2 cell differentiation	7	2.60E-03
		AGE-RAGE signaling pathway in diabetic complications	6	2.80E-02
		Parathyroid hormone synthesis, secretion and action	6	3.10E-02
		Inflammatory bowel disease	5	3.10E-02
		Chemical carcinogenesis - receptor activation	8	3.10E-02
		Acute myeloid leukemia	5	3.10E-02
		Non-small cell lung cancer	5	3.80E-02
		Pancreatic cancer	5	4.30E-02
		Epstein-Barr virus infection	7	7.90E-02
		Prostate cancer	5	8.70E-02
		Endocrine resistance	5	8.70E-02
		Cellular senescence	6	9.30E-02
		Hepatocellular carcinoma	6	1.20E-01
		Wnt signaling pathway	6	1.20E-01
		Growth hormone synthesis, secretion, and action	5	1.40E-01
		FoxO signaling pathway	5	1.80E-01
		Measles	5	2.10E-01
		Signaling pathways regulating pluripotency of stem cells	5	2.20E-01
		Gastric cancer	5	2.40E-01

		Small cell lung cancer	4	2.50E-01
		Cushing syndrome	5	2.50E-01
		Human cytomegalovirus infection	6	2.50E-01
		Thyroid hormone signaling pathway	4	4.30E-01
		Kaposi sarcoma-associated herpes virus infection	5	4.30E-01
		Notch signaling pathway	3	4.30E-01
SH2D1B	10	Progesterone-mediated oocyte maturation	2	5.20E-01
		Cell cycle	2	5.20E-01
		Oocyte meiosis	2	5.20E-01
		Gastric cancer	2	5.20E-01
		Cellular senescence	2	5.20E-01
		Hepatocellular carcinoma	2	5.20E-01
INPP5F	109	Transcriptional misregulation in cancer	15	3.40E-09
		Non-small cell lung cancer	4	4.50E-01
		Chemical carcinogenesis - receptor activation	6	4.50E-01
		Human T-cell leukemia virus 1 infection	6	4.50E-01
		Breast cancer	5	4.50E-01
		Thermogenesis	6	4.50E-01
		Hepatocellular carcinoma	5	5.20E-01
		Parathyroid hormone synthesis, secretion, and action	4	5.80E-01
		Viral carcinogenesis	5	6.60E-01

		Herpes simplex virus 1 infection	8	6.60E-01
		Estrogen signaling pathway	4	8.10E-01
		Spliceosome	4	8.10E-01
		Mitophagy - animal	3	8.10E-01
		Hippo signaling pathway	4	8.10E-01
		Chronic myeloid leukemia	3	8.10E-01
		Hepatitis B	4	8.10E-01
INPP5K	4	-	-	-
**PSMA1 paralogs**				
PSMA8	61	Herpes simplex virus 1 infection	18	6.40E-13
PSMB1	35	Herpes simplex virus 1 infection	13	3.80E-11
PSMA5	64	Herpes simplex virus 1 infection	15	1.70E-09
		Maturity onset diabetes of the young	3	3.70E-02
PSMB7	43	Herpes simplex virus 1 infection	13	6.00E-09
		Transcriptional misregulation in cancer	3	1.00E+00
PSMB3	5	-	-	-
**USH2A paralog**				
ATRNL1	42	Breast cancer	6	9.50E-03
		Chemical carcinogenesis–receptor activation	6	2.60E-02
		Pathways in cancer	8	3.70E-02
		Kaposi sarcoma-associated herpes virus infection	5	7.60E-02
		Maturity onset diabetes of the young	3	7.60E-02
		Th17 cell differentiation	4	8.30E-02

		Human T-cell leukemia virus 1 infection	5	8.30E-02
		Hepatitis B	4	2.20E-01
		Renal cell carcinoma	3	2.80E-01
		ErbB signaling pathway	3	3.70E-01
		Th1 and Th2 cell differentiation	3	3.70E-01
		Prostate cancer	3	3.70E-01
		Endocrine resistance	3	3.70E-01
		HIF-1 signaling pathway	3	4.20E-01
		Growth hormone synthesis, secretion, and action	3	4.40E-01
		Thyroid hormone signaling pathway	3	4.40E-01
		Estrogen signaling pathway	3	5.20E-01
		Human papilloma virus infection	4	5.90E-01
		JAK-STAT signaling pathway	3	6.20E-01
		Hepatocellular carcinoma	3	6.30E-01
**MACROD2 paralog**				
MACROD1 (ERV1-equivalent)	2	Necroptosis	2	3.50E-01
MACROD1 (ERV2-equivalent)	5	Transcriptional misregulation in cancer	2	1.00E+00

*Adjusted p-values by using the linear step-up method by Benjamini and Hochberg

Table S13. Cosmic variants identified within sections with high expression levels in brain tissues.

**Gen associated**	**Cosmic variant ID**	**Phenotype**
INPP5B	COSV65961705	Liver tumor
INPP5B	COSV65961588	Breast tumor
TTLL5	COSV54041222	Biliary tract tumor
CENPC	COSV56625135	Central nervous system tumor
KLHL24	COSV54428449	Large intestine tumor
POLDIP3	COSV52808748	Breast tumor; Esophageal tumor
POLDIP3	COSV52808459	Breast tumor
POLDIP3	COSV52808465	Breast tumor

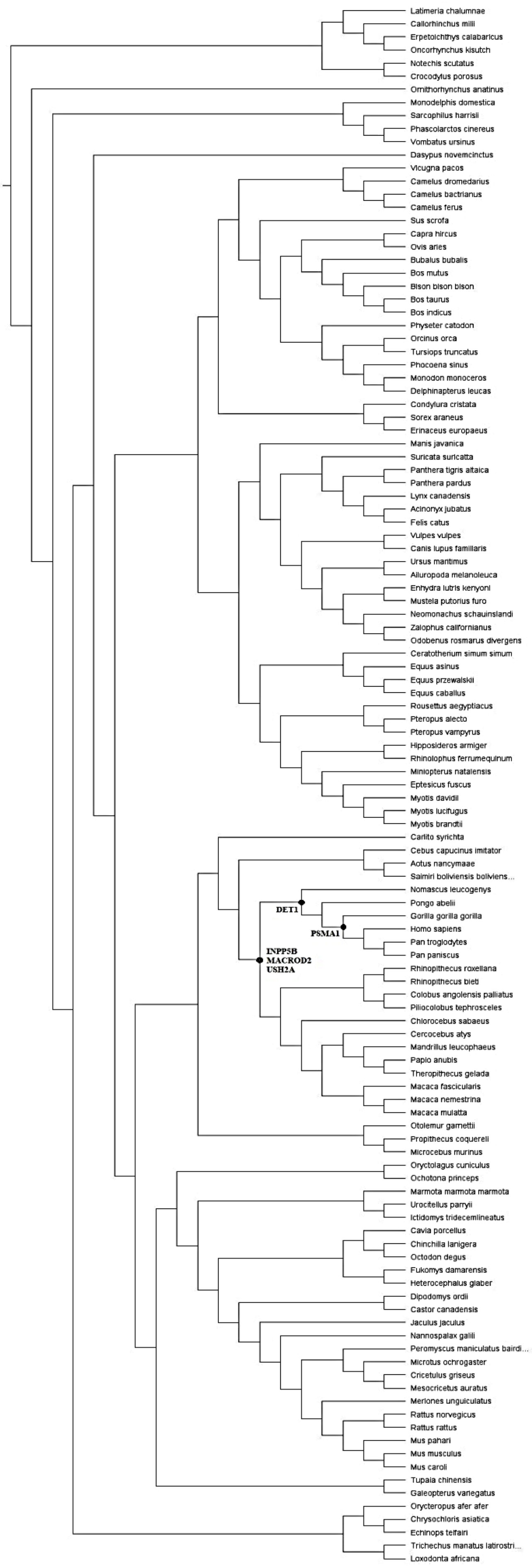

Fig S1. Mammal species tree with the location of the minimum common ancestor where the retrovirus infection occurred for the genes analyzed in this work.

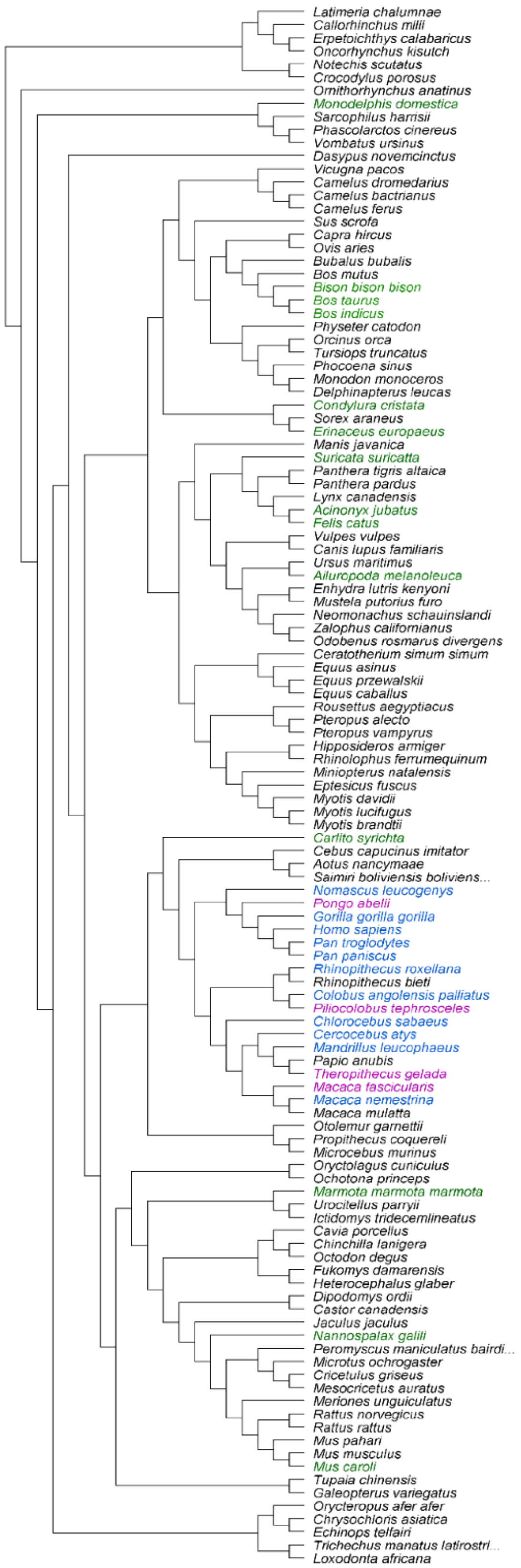

Fig S2. Mammal species tree with the location of the minimum common ancestor where the retrovirus infection occurred for the *INPP5B* gene. Blue represents the species where ERVs were detected with both RetroTector and BLASTN, purple for species detected only by alignment with BLASTN, and green for species detected by RetroTector only.

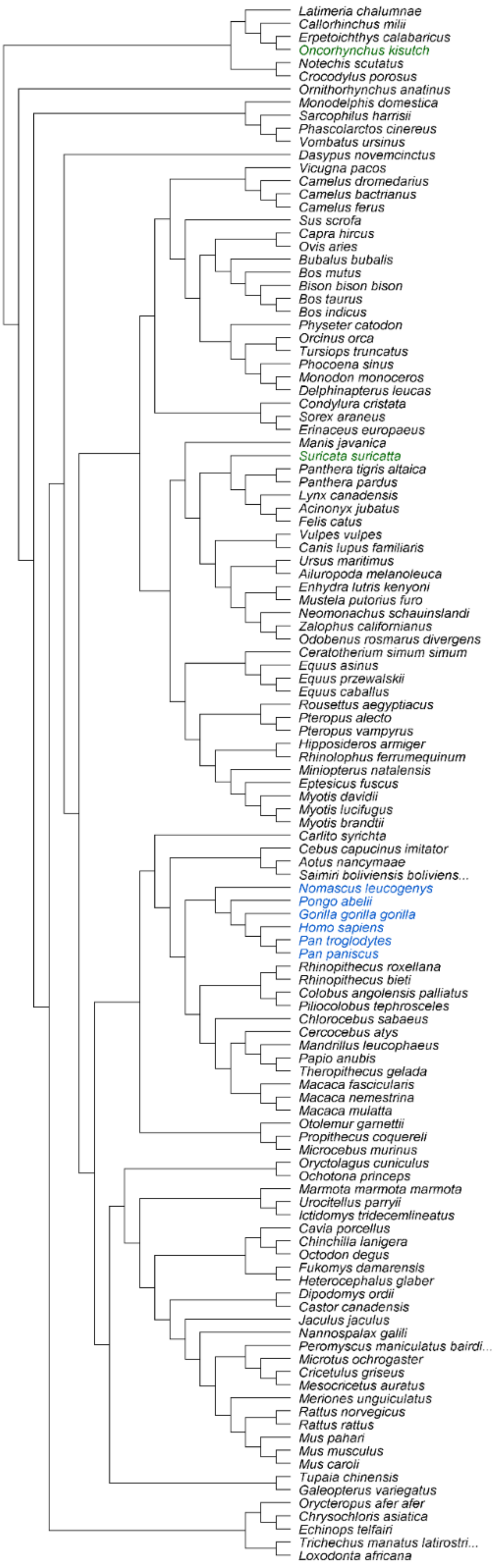

Fig S3. Mammal species tree with the location of the minimum common ancestor where the retrovirus infection occurred for the *DET1* gene. Blue represents the species where ERVs were

detected with both RetroTector and BLASTN, purple for species detected only by alignment with BLASTN, and green for species detected by RetroTector only.

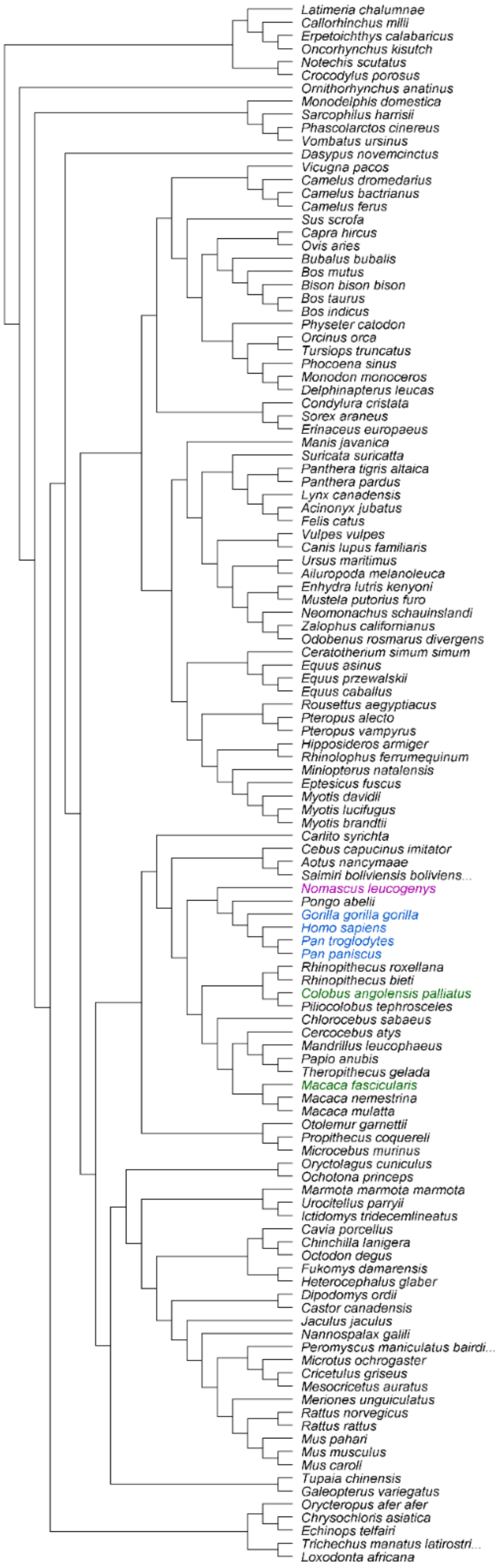

Fig S4. Mammal species tree with the location of the minimum common ancestor where the retrovirus infection occurred for the PSMA1 gene. Blue represents the species where ERVs were detected with both RetroTector and BLASTN, purple for species detected only by alignment with BLASTN, and green for species detected by RetroTector only.

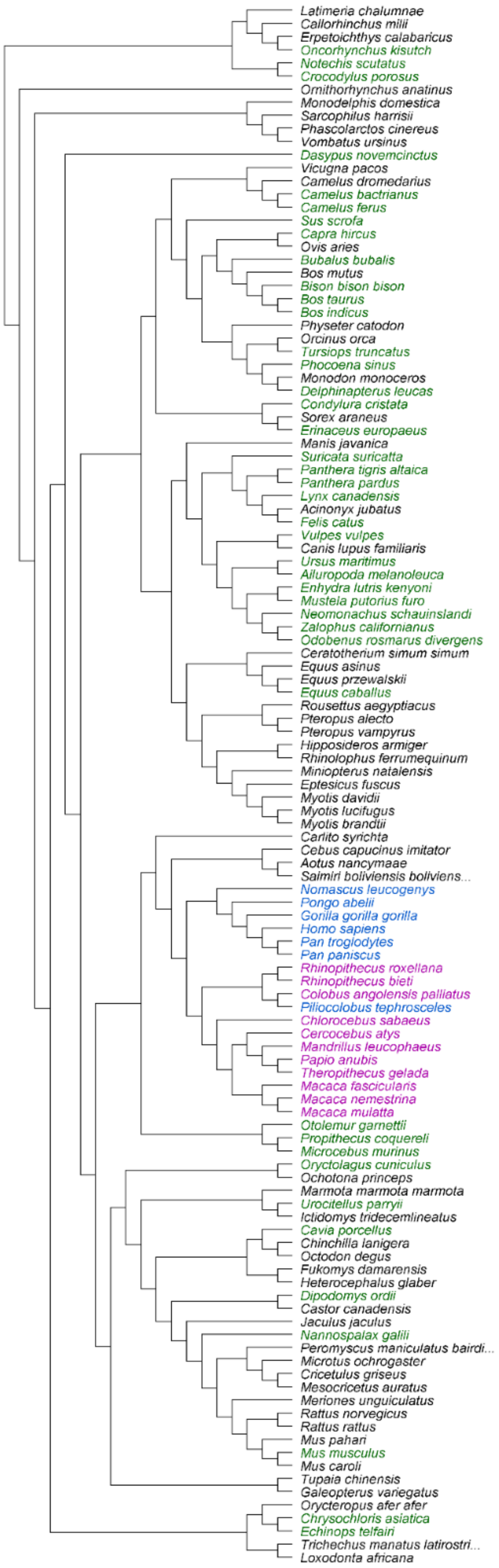

Fig S5. Mammal species tree with the location of the minimum common ancestor where the retrovirus infection occurred for the *USH2A* gene. Blue represents the species where ERVs

were detected with both RetroTector and BLASTN, purple for species detected only by alignment with BLASTN, and green for species detected by RetroTector only.

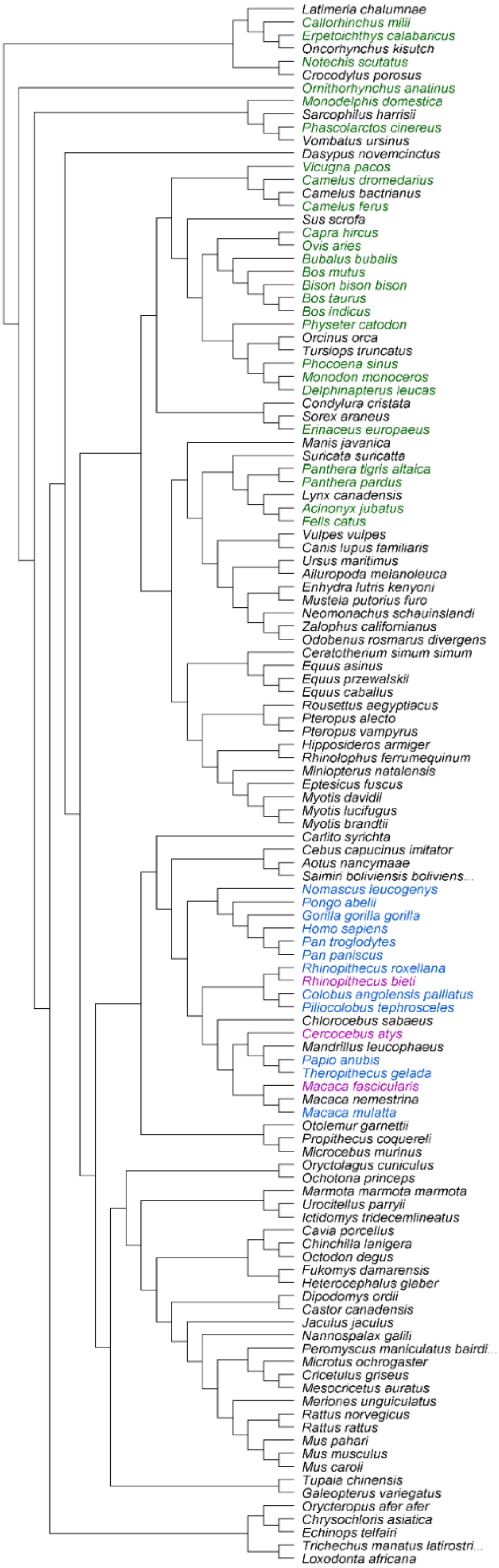

Fig S6. Mammal species tree with the location of the minimum common ancestor where the retrovirus infection occurred for the *MACROD2* gene. Blue represents the species where ERVs were detected with both RetroTector and BLASTN, purple for species detected only by alignment with BLASTN, and green for species detected by RetroTector only.
