# Supplementary material for: Evolutionary analysis of endogenous intronic retroviruses in primates reveals an enrichment in transcription binding sites associated with key regulatory processes
Source: PeerJ. 2022 Dec 22;10:e14431. doi: 10.7717/peerj.14431 (PMC9790151; doi:10.7717/peerj.14431)

Supplementary file 2. Expression of the putative lncRNA inside human gene introns and their annotation by RepeatMasker (data extracted from UCSC browser). Brain hippocampus expression was used as brain tissues reference.

TTLL5

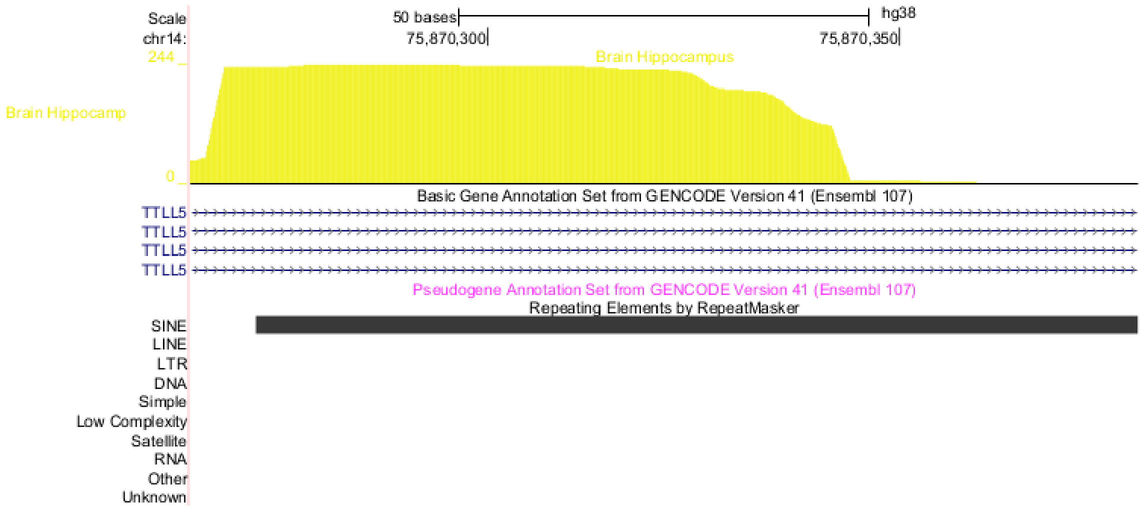

CENPC

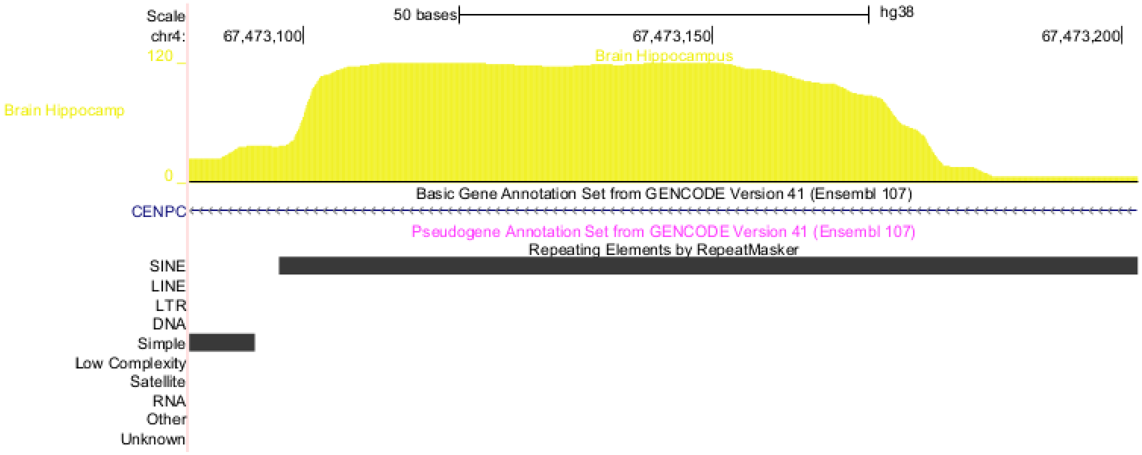

KLHL24

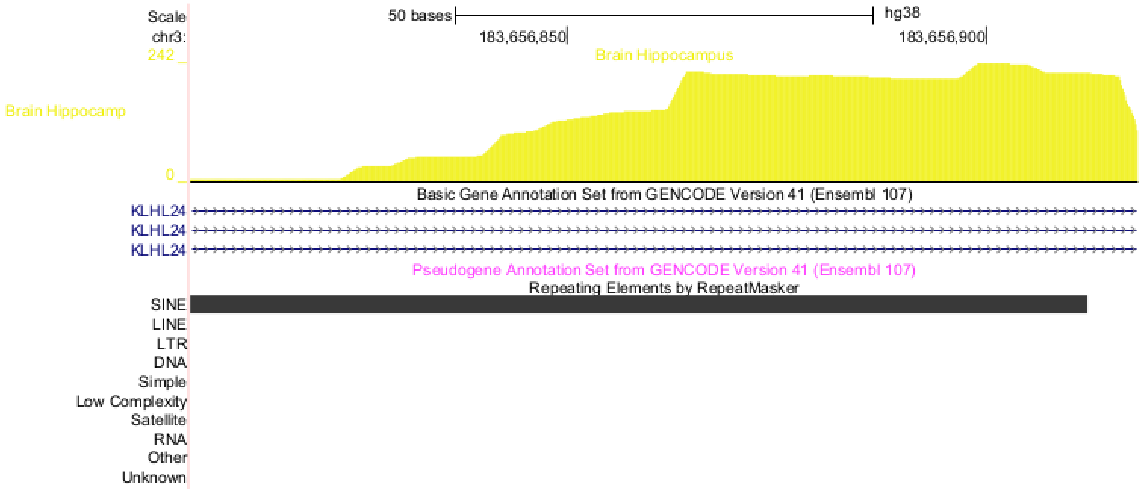

DPYLS2

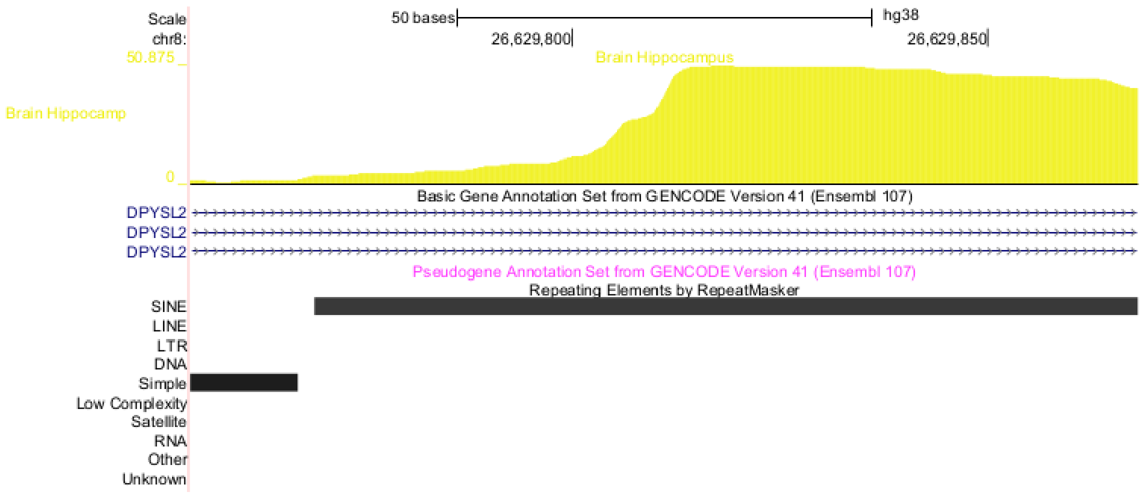

PICALM

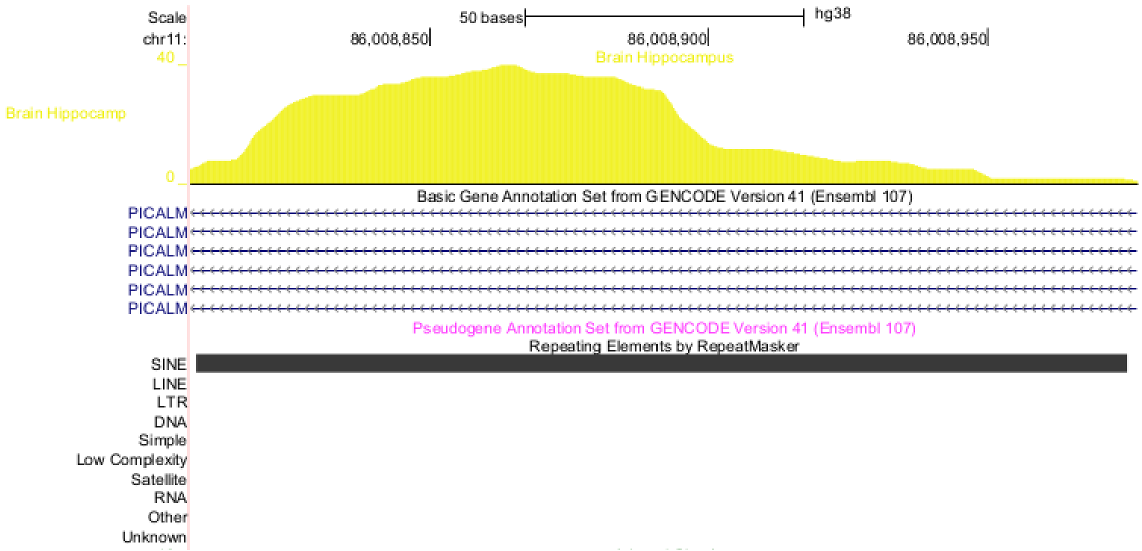

PVT1

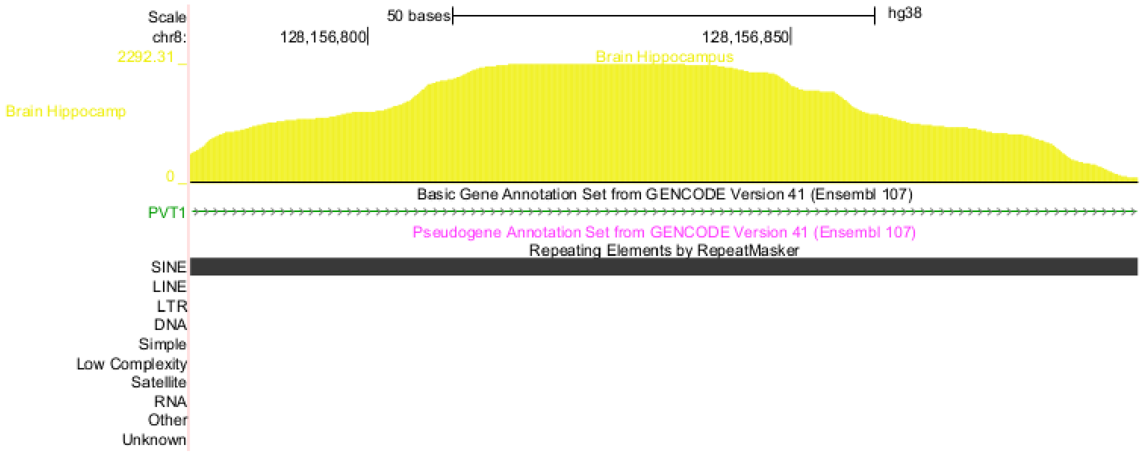

EPCAM-DT

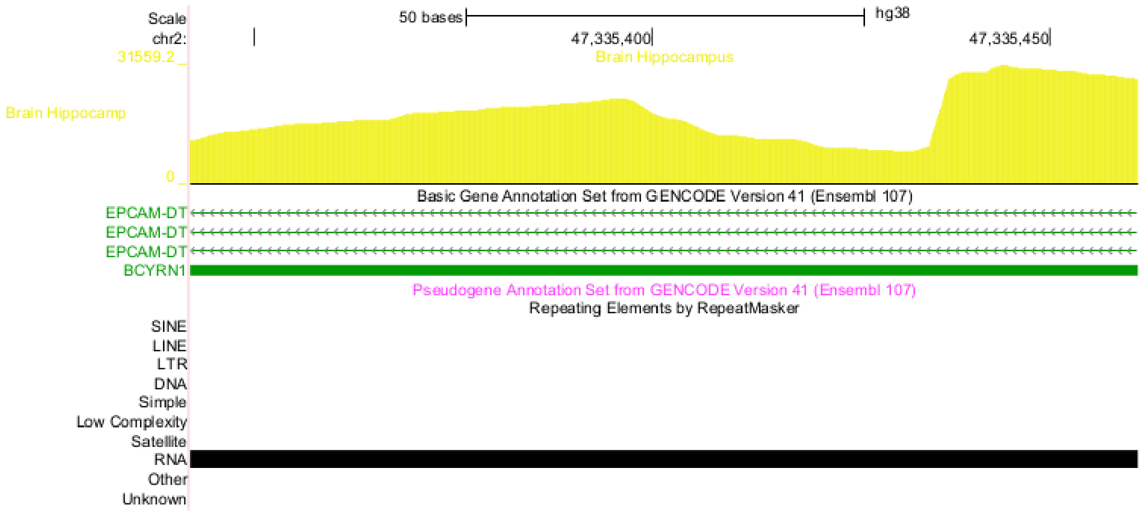

MEF2C-AS1

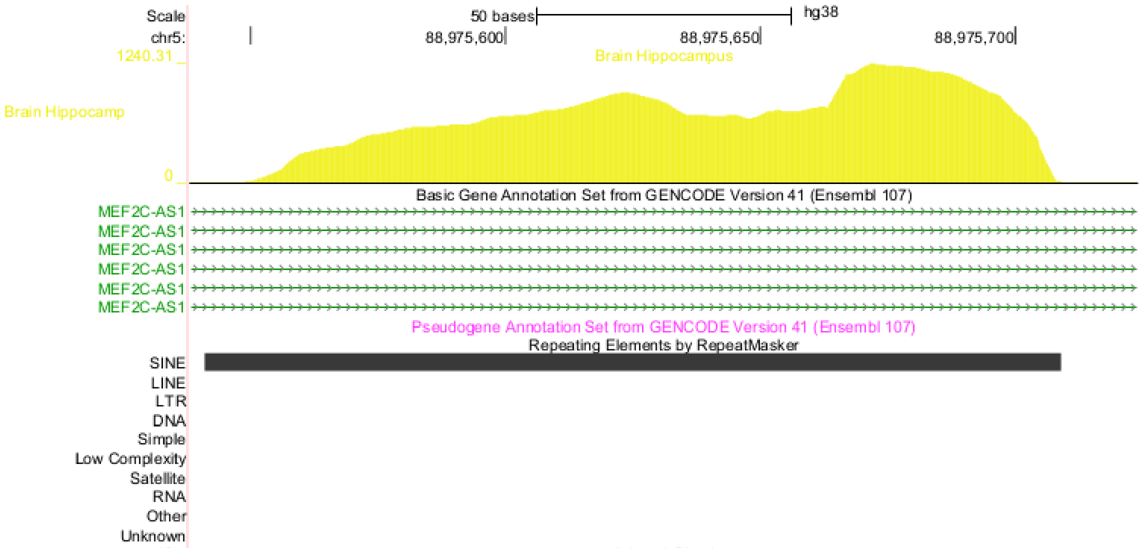

POLDIP3

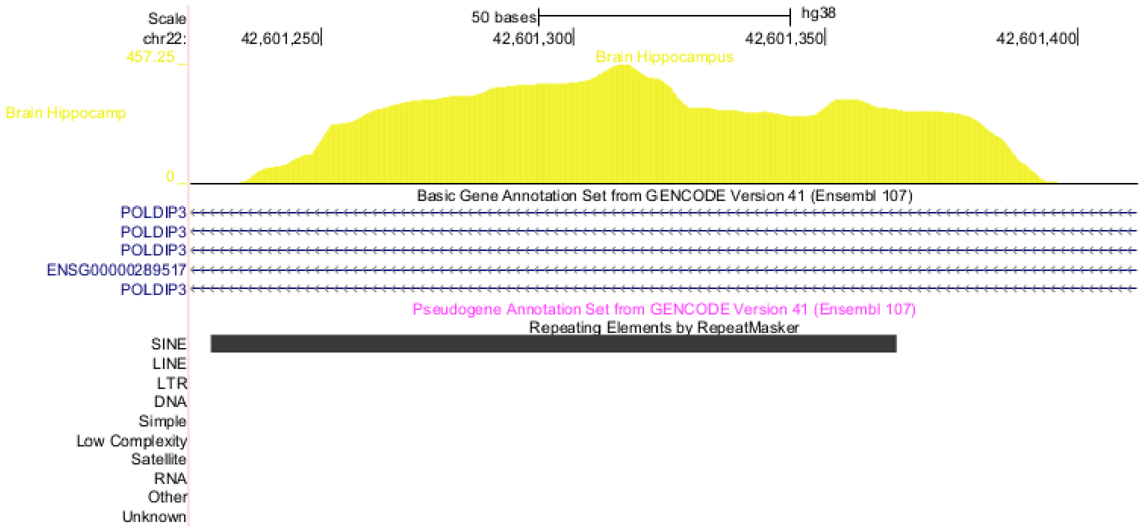

Supplement: File S2 — Brain hippocampus expression was used as brain tissue reference. [file peerj-10-14431-s002.pdf]
