# Supplementary material for: Evolutionary analysis of endogenous intronic retroviruses in primates reveals an enrichment in transcription binding sites associated with key regulatory processes
Source: PeerJ. 2022 Dec 22;10:e14431. doi: 10.7717/peerj.14431 (PMC9790151; doi:10.7717/peerj.14431)
Supplement: File S3 — Data visualized in UCSC Genome Browser (http://genome.ucsc.edu) using the GTEx portal data (https://gtexportal.org). [file peerj-10-14431-s003.pdf]

Supplementary file 3. ERV expression in several tissues. Data visualized in UCSC genome browser (http://genome.ucsc.edu) using the GTEx portal data (https://gtexportal.org/)

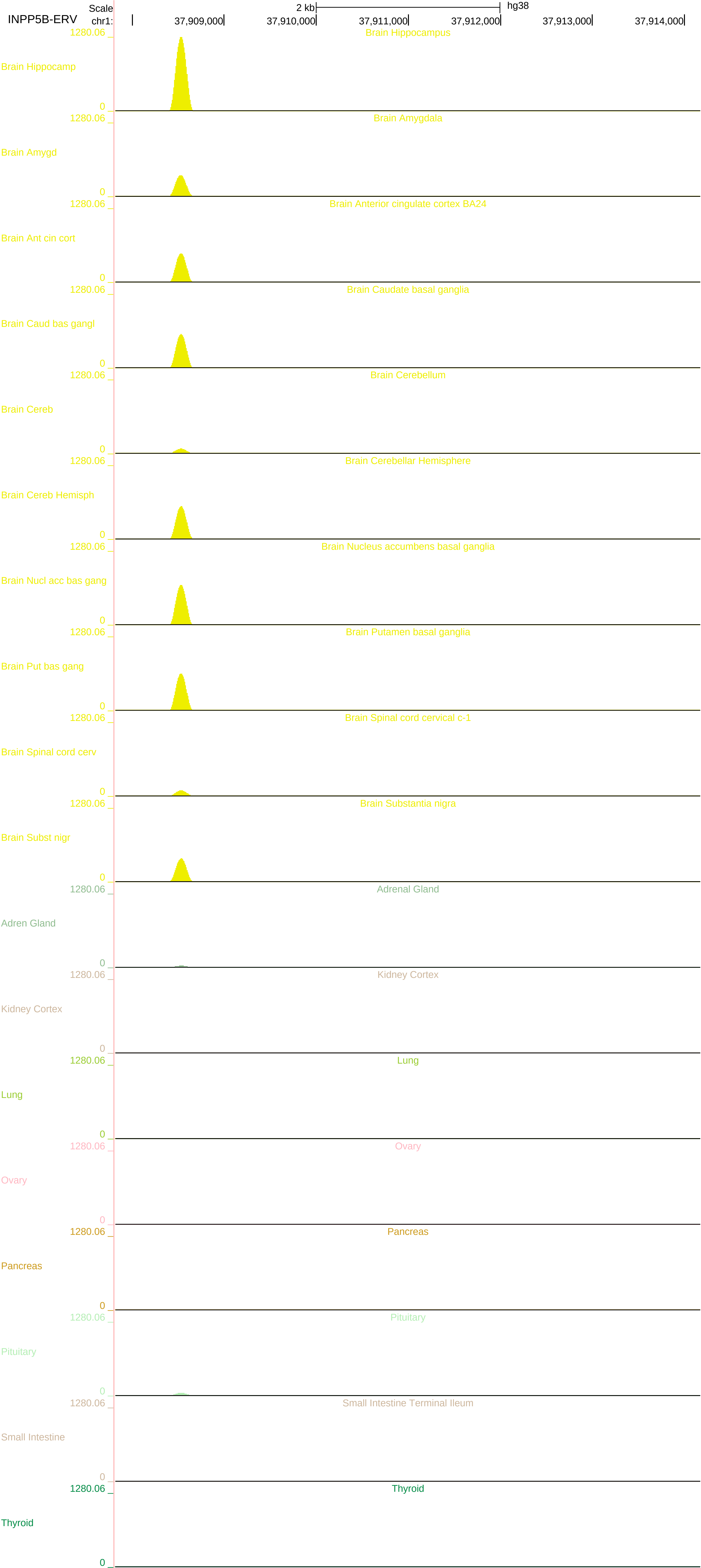
INPP5B-ERV
Scale
chr1:
2 kb
hg38
37,909,000
37,910,000
37,911,000
37,912,000
37,913,000
37,914,000
Brain Hippocampus
Brain Hippocamp
Brain Amygdala
Brain Amygd
Brain Anterior cingulate cortex BA24
Brain Ant cin cort
Brain Caudate basal ganglia
Brain Caud bas gangl
Brain Cerebellum
Brain Cereb
Brain Cerebellar Hemisphere
Brain Cereb Hemisph
Brain Nucleus accumbens basal ganglia
Brain Nucl acc bas gang
Brain Putamen basal ganglia
Brain Put bas gang
Brain Spinal cord cervical c-1
Brain Spinal cord cerv
Brain Substantia nigra
Brain Subst nigr
Adrenal Gland
Adren Gland
Kidney Cortex
Lung
Ovary
Pancreas
Pituitary
Small Intestine Terminal Ileum
Small Intestine
Thyroid
1280.06
0

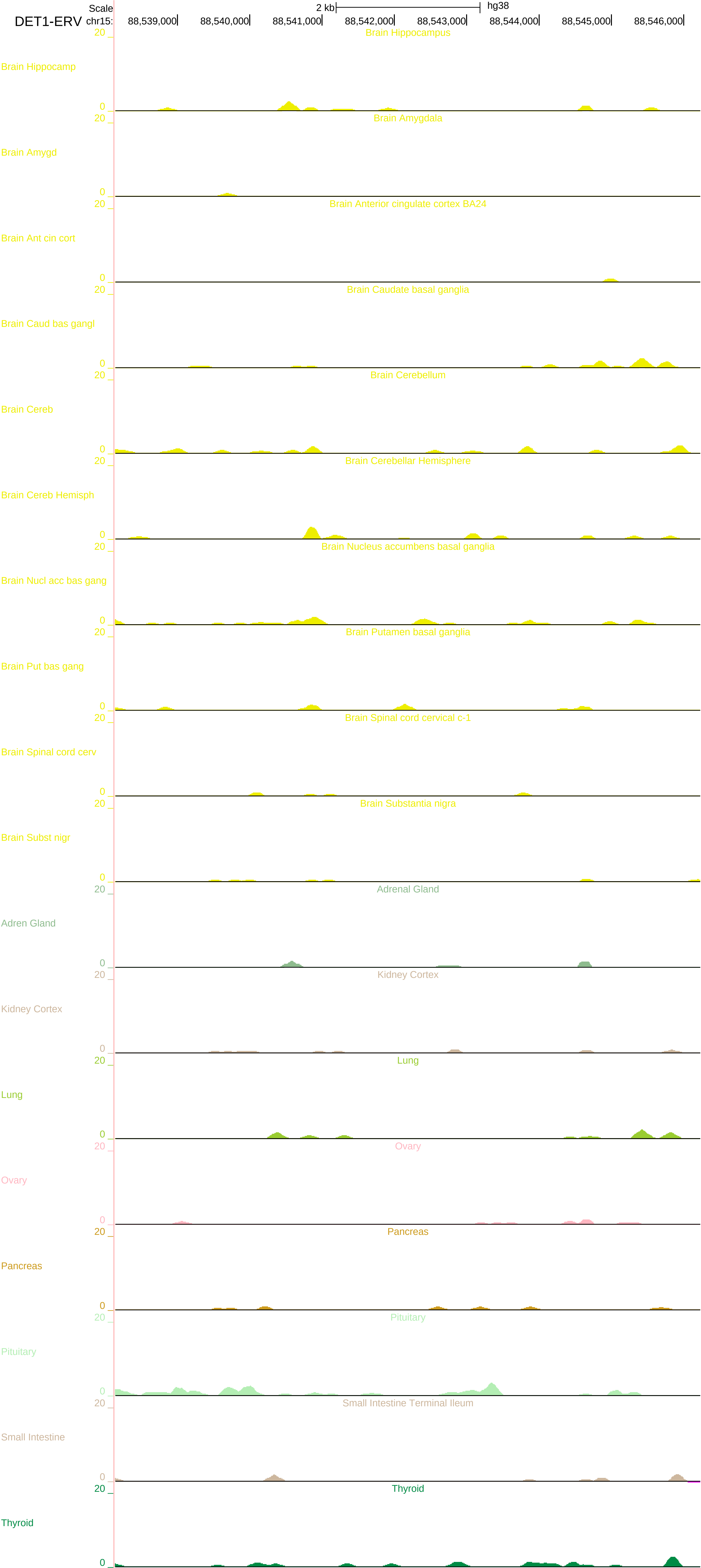
DET1-ERV
Scale
2 kb
hg38
chr15:
88,539,000
88,540,000
88,541,000
88,542,000
88,543,000
88,544,000
88,545,000
88,546,000
Brain Hippocampus
Brain Hippocamp
Brain Amygdala
Brain Amygd
Brain Anterior cingulate cortex BA24
Brain Ant cin cort
Brain Caudate basal ganglia
Brain Caud bas gangl
Brain Cerebellum
Brain Cereb
Brain Cerebellar Hemisphere
Brain Cereb Hemisph
Brain Nucleus accumbens basal ganglia
Brain Nucl acc bas gang
Brain Putamen basal ganglia
Brain Put bas gang
Brain Spinal cord cervical c-1
Brain Spinal cord cerv
Brain Substantia nigra
Brain Subst nigr
Adrenal Gland
Adren Gland
Kidney Cortex
Lung
Ovary
Pancreas
Pituitary
Small Intestine Terminal Ileum
Small Intestine
Thyroid
20
0

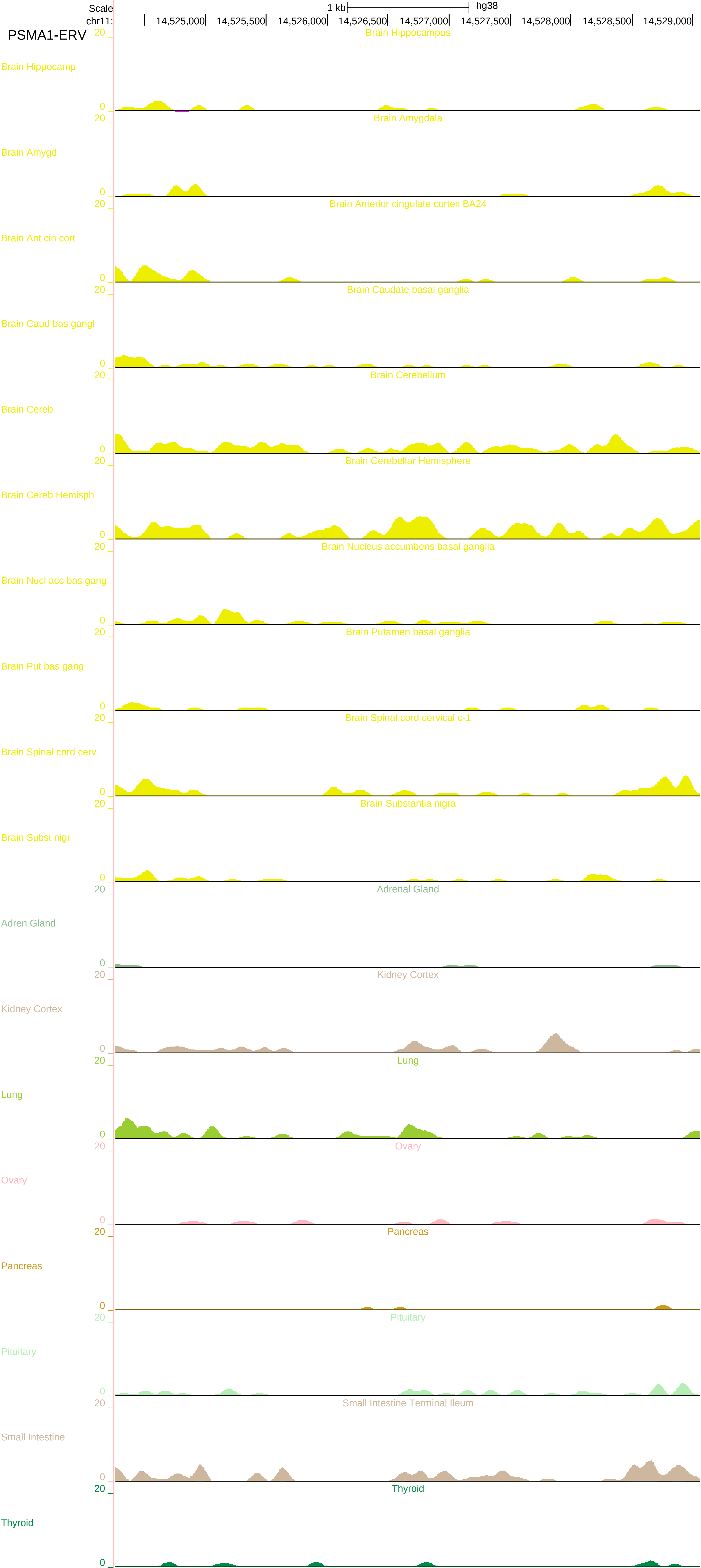
Scale
chr11:
1 kb
hg38
14,525,000
14,525,500
14,526,000
14,526,500
14,527,000
14,527,500
14,528,000
14,528,500
14,529,000
PSMA1-ERV
Brain Hippocampus
Brain Hippocamp
Brain Amygdala
Brain Amygd
Brain Anterior cingulate cortex BA24
Brain Ant cin cort
Brain Caudate basal ganglia
Brain Caud bas gangl
Brain Cerebellum
Brain Cereb
Brain Cerebellar Hemisphere
Brain Cereb Hemisph
Brain Nucleus accumbens basal ganglia
Brain Nucl acc bas gang
Brain Putamen basal ganglia
Brain Put bas gang
Brain Spinal cord cervical c-1
Brain Spinal cord cerv
Brain Substantia nigra
Brain Subst nigr
Adrenal Gland
Adren Gland
Kidney Cortex
Lung
Ovary
Pancreas
Pituitary
Small Intestine Terminal Ileum
Small Intestine
Thyroid
20
0

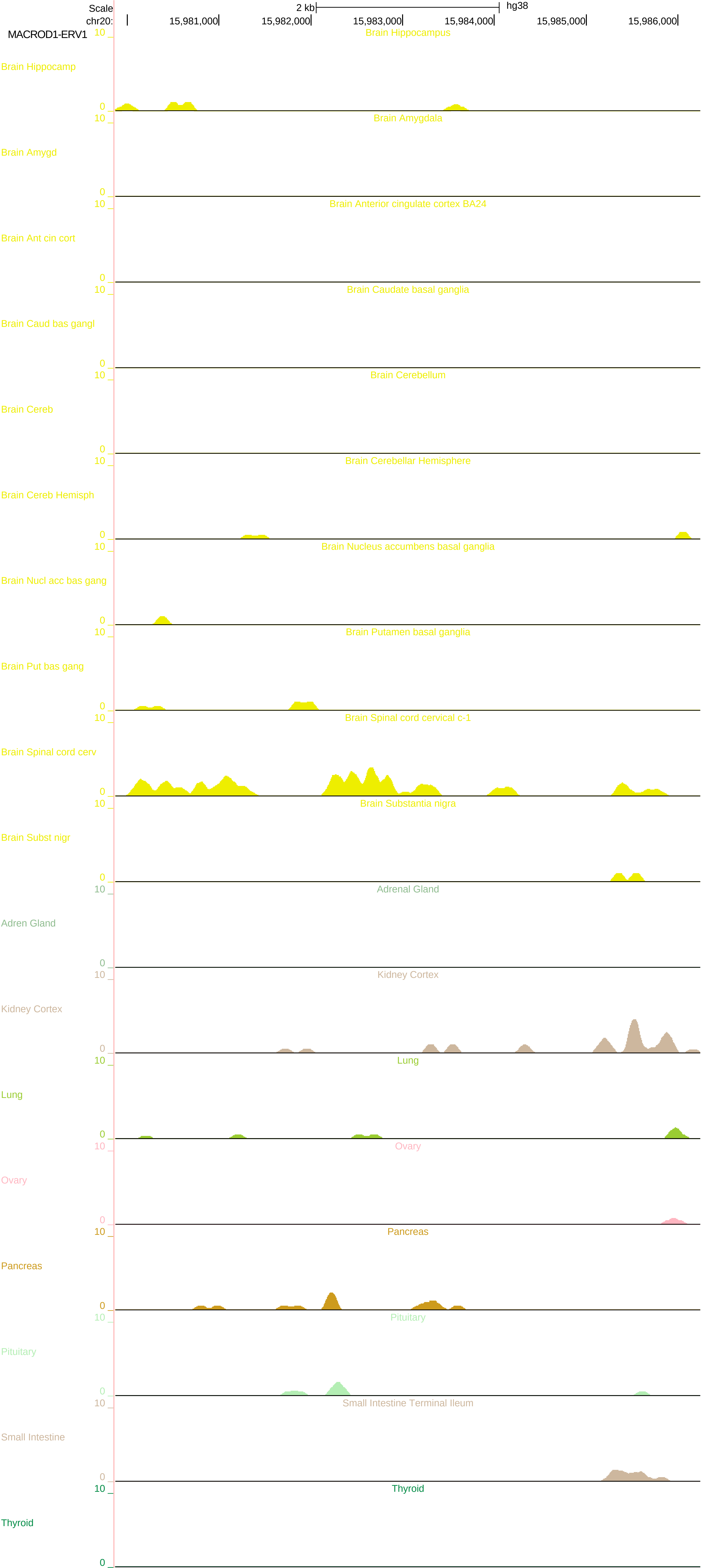
Scale
2 kb
hg38
chr20:
15,981,000
15,982,000
15,983,000
15,984,000
15,985,000
15,986,000
MACROD1-ERV1
Brain Hippocampus
Brain Hippocamp
Brain Amygdala
Brain Amygd
Brain Anterior cingulate cortex BA24
Brain Ant cin cort
Brain Caudate basal ganglia
Brain Caud bas gangl
Brain Cerebellum
Brain Cereb
Brain Cerebellar Hemisphere
Brain Cereb Hemisph
Brain Nucleus accumbens basal ganglia
Brain Nucl acc bas gang
Brain Putamen basal ganglia
Brain Put bas gang
Brain Spinal cord cervical c-1
Brain Spinal cord cerv
Brain Substantia nigra
Brain Subst nigr
Adrenal Gland
Adren Gland
Kidney Cortex
Kidney Cortex
Lung
Lung
Ovary
Ovary
Pancreas
Pancreas
Pituitary
Pituitary
Small Intestine Terminal Ileum
Small Intestine
Thyroid
Thyroid
10
0

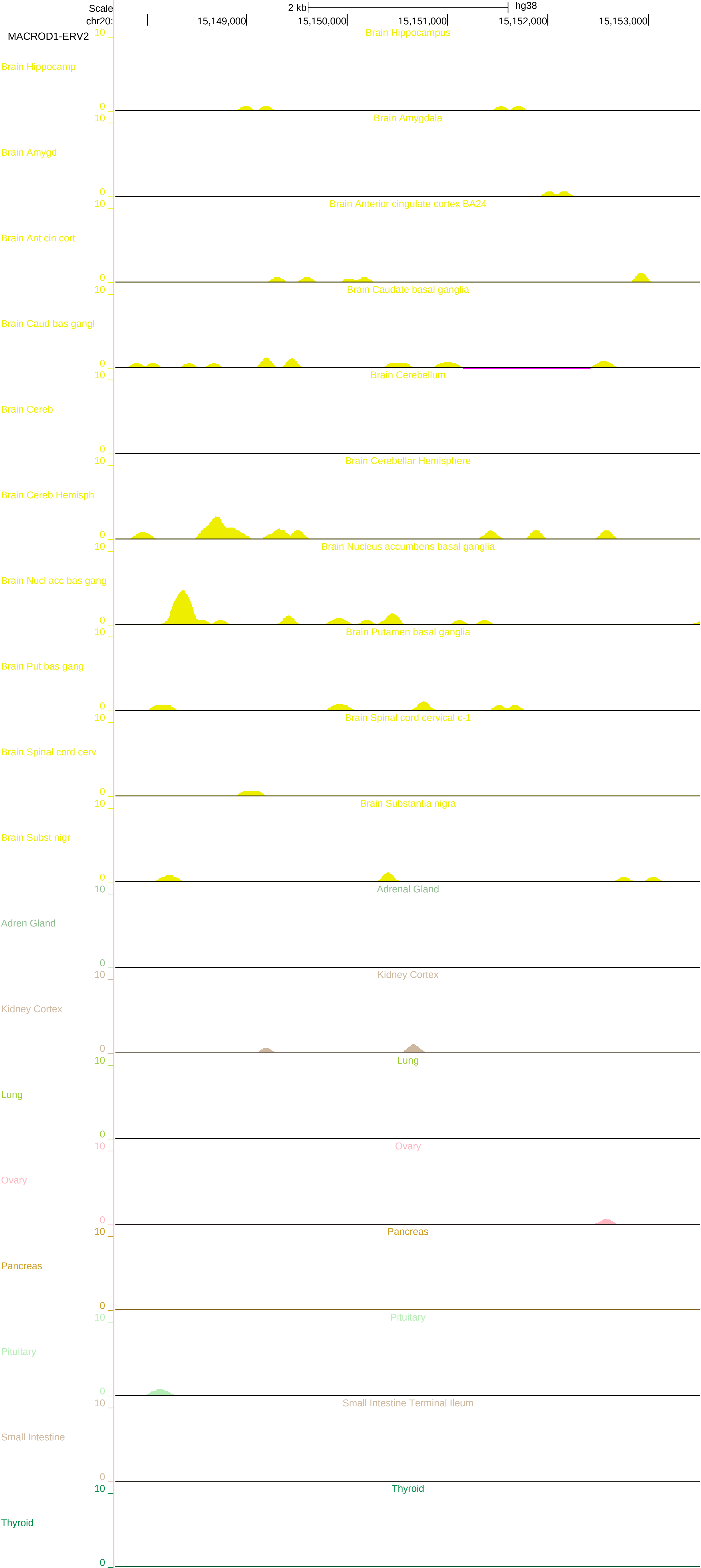
Scale
2 kb
hg38
chr20:
15,149,000
15,150,000
15,151,000
15,152,000
15,153,000
MACROD1-ERV2
Brain Hippocampus
Brain Hippocamp
Brain Amygdala
Brain Amygd
Brain Anterior cingulate cortex BA24
Brain Ant cin cort
Brain Caudate basal ganglia
Brain Caud bas gangl
Brain Cerebellum
Brain Cereb
Brain Cerebellar Hemisphere
Brain Cereb Hemisph
Brain Nucleus accumbens basal ganglia
Brain Nucl acc bas gang
Brain Putamen basal ganglia
Brain Put bas gang
Brain Spinal cord cervical c-1
Brain Spinal cord cerv
Brain Substantia nigra
Brain Subst nigr
Adrenal Gland
Adren Gland
Kidney Cortex
Lung
Ovary
Pancreas
Pituitary
Small Intestine Terminal Ileum
Small Intestine
Thyroid
10
0

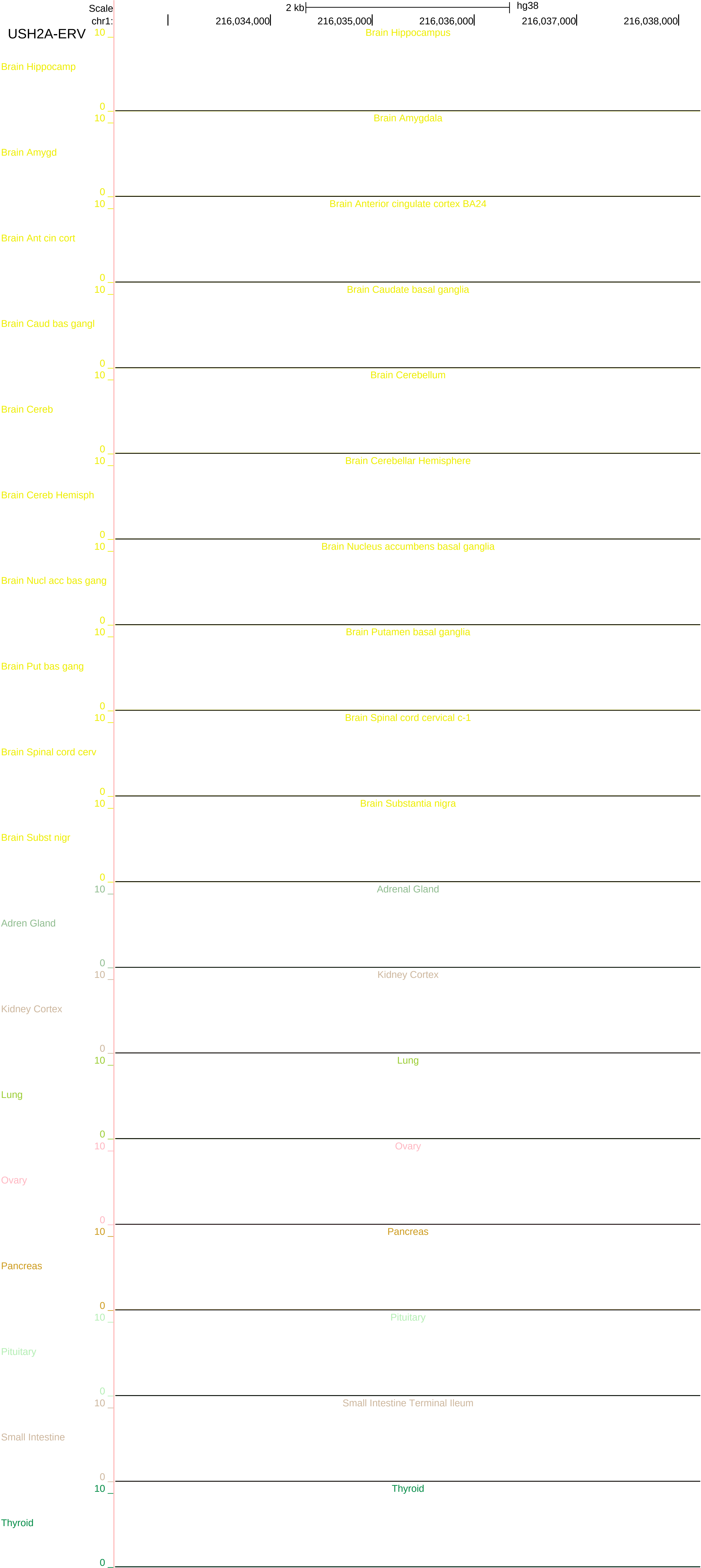

Scale
chr1:
2 kb
hg38
216,034,000
216,035,000
216,036,000
216,037,000
216,038,000
USH2A-ERV
Brain Hippocampus
Brain Hippocamp
Brain Amygdala
Brain Amygd
Brain Anterior cingulate cortex BA24
Brain Ant cin cort
Brain Caudate basal ganglia
Brain Caud bas gangl
Brain Cerebellum
Brain Cereb
Brain Cerebellar Hemisphere
Brain Cereb Hemisph
Brain Nucleus accumbens basal ganglia
Brain Nucl acc bas gang
Brain Putamen basal ganglia
Brain Put bas gang
Brain Spinal cord cervical c-1
Brain Spinal cord cerv
Brain Substantia nigra
Brain Subst nigr
Adrenal Gland
Adren Gland
Kidney Cortex
Lung
Ovary
Pancreas
Pituitary
Small Intestine Terminal Ileum
Small Intestine
Thyroid
10
0
